# Supplementary material for: Association between epidural catheter tip malposition and anesthesiologists’ experience after graduation: A cross-sectional study using postoperative CT images
Source: PLoS One. 2025 Jun 26;20(6):e0316304. doi: 10.1371/journal.pone.0316304 (PMC12200878; doi:10.1371/journal.pone.0316304)
Supplement: S1 Table — (DOCX) [file pone.0316304.s001.docx]

**S1 Table. Sensitivity analysis excluding one anesthesiologist who performed the most epidural anesthesia procedures and had the highest number of malpositions**

|  | Normal position (n=156) | Malposition (n=20) | P value |
| --- | --- | --- | --- |
| Patient age, years | 71 [17, 89] | 71 [27, 86] | 0.598 |
| Patient sex, female | 65 (42%) | 8 (40%) | >0.999 |
| Patient body mass index, kg/m^2^ | 23.0 [14.9, 35.2] | 21.8 [13.6, 29.0] | 0.517 |
| Vertebral level  T4/5/6/7/8/9/10/11/12/L1/2/3 | 1/1/4/30/41/36/  20/13/8/2/0/0 | 0/0/2/2/4/4/  2/3/2/1/0/0 | 0.921 |
| Anesthesiologists’ experience, years | 5.3 [2.0, 35.4] | 11.4 [2.1, 26.6] | 0.006 |
| Anesthesiologist sex, female | 93 (60%) | 13 (65%) | 0.809 |
| Postoperative day 0/1/2/3/4/5 | 0/15/29/72/38/2 | 0/3/4/10/3/0 | 0.286 |
| Length of epidural catheter advanced after LOR, cm | 5.0 [3.0, 7.0] | 5.0 [4.0, 6.0] | 0.297 |

The data are presented as frequencies (%) and medians [range]. Postoperative day indicates the day the CT image was taken. Comparisons between groups were conducted using the chi-square and Mann-Whitney U tests. LOR, loss-of-resistance.
