# Supplementary material for: Association between epidural catheter tip malposition and anesthesiologists’ experience after graduation: A cross-sectional study using postoperative CT images
Source: PLoS One. 2025 Jun 26;20(6):e0316304. doi: 10.1371/journal.pone.0316304 (PMC12200878; doi:10.1371/journal.pone.0316304)
Supplement: S2 Table — (DOCX) [file pone.0316304.s002.docx]

**S2 Table. Sensitivity analysis excluding 37 epidural anesthesia procedures performed by anesthesiologists with 2.0-2.5 years’ experience.**

|  | Normal position (n=1  30) | Malposition (n=22) | P value |
| --- | --- | --- | --- |
| Patient age, years | 71 [15, 89] | 70 [27, 86] | 0.575 |
| Patient sex, female | 57 (44%) | 7 (32%) | 0.355 |
| Patient body mass index, kg/m^2^ | 22.5 [14.9, 35.2] | 21.3 [13.6, 31.3] | 0.420 |
| Vertebral level  T4/5/6/7/8/9/10/11/12/L1/2/3 | 1/2/4/23/38/32/  16/8/4/2/0/0 | 0/0/2/2/3/3/  2/5/2/2/0/1 | 0.012 |
| Anesthesiologists’ experience, years | 7.0 [2.5, 35.4] | 11.4 [2.7, 26.6] | 0.037 |
| Anesthesiologist sex, female | 73 (56%) | 12 (55%) | >0.999 |
| Postoperative day 0/1/2/3/4/5 | 0/15/27/49/37/2 | 0/3/4/12/3/0 | 0.358 |
| Length of epidural catheter advanced after LOR, cm | 5.0 [3.0, 7.0] | 5.0 [4.0, 6.0] | 0.745 |

The data are presented as frequencies (%) and medians [range]. Postoperative day indicates the day the CT image was taken. Comparisons between groups were conducted using the chi-square and Mann-Whitney U tests. LOR, loss-of-resistance.
