# Supplementary material for: Association between epidural catheter tip malposition and anesthesiologists’ experience after graduation: A cross-sectional study using postoperative CT images
Source: PLoS One. 2025 Jun 26;20(6):e0316304. doi: 10.1371/journal.pone.0316304 (PMC12200878; doi:10.1371/journal.pone.0316304)
Supplement: S3 Table — (DOCX) [file pone.0316304.s003.docx]

**S3 Table. Sensitivity analysis excluding six epidural anesthesia procedures performed by anesthesiologists with more than 20 years of postgraduate experience.**

|  | Normal position (n=161) | Malposition (n=22) | P value |
| --- | --- | --- | --- |
| Patient age, years | 71 [15, 89] | 70 [27, 86] | 0.548 |
| Patient sex, female | 66 (41%) | 8 (36%) | 0.818 |
| Patient body mass index, kg/m^2^ | 22.7 [14.9, 35.2] | 21.1 [13.6, 31.3] | 0.136 |
| Vertebral level  T4/5/6/7/8/9/10/11/12/L1/2/3 | 1/2/3/31/43/37/  21/13/8/2/0/0 | 0/0/1/1/4/4/  2/5/2/2/0/1 | 0.074 |
| Anesthesiologists’ experience, years | 5.6 [2.0, 19.6] | 8.8 [2.1, 18.6] | 0.024 |
| Anesthesiologist sex, female | 91 (57%) | 11 (50%) | 0.649 |
| Postoperative day 0/1/2/3/4/5 | 0/17/29/73/40/2 | 0/3/4/12/3/0 | 0.331 |
| Length of epidural catheter advanced after LOR, cm | 5.0 [3.0, 7.0] | 5.0 [4.0, 6.0] | 0.562 |

The data are presented as frequencies (%) and medians [range]. Postoperative day indicates the day the CT image was taken. Comparisons between groups were conducted using the chi-square and Mann-Whitney U tests. LOR, loss-of-resistance.
