# Supplementary material for: Association between epidural catheter tip malposition and anesthesiologists’ experience after graduation: A cross-sectional study using postoperative CT images
Source: PLoS One. 2025 Jun 26;20(6):e0316304. doi: 10.1371/journal.pone.0316304 (PMC12200878; doi:10.1371/journal.pone.0316304)
Supplement: Attachment — Submitted filename: Response to Reviewers.docx [file pone.0316304.s006.docx]

**Response to Reviewers**

**Manuscript ID:** PONE-D-24-56351
**Title:** *Association between epidural catheter tip malposition and anesthesiologists’ experience after graduation: a cross-sectional study using postoperative CT images*

Dear Dr. Alessandro De Cassai and Reviewers,

We would like to express our sincere appreciation for your valuable feedback and the opportunity to revise our manuscript. We carefully reviewed each comment and revised the manuscript accordingly. We believe that the quality and clarity of the manuscript have improved substantially through this process.

Below, we provide our point-by-point responses to all reviewer and editorial comments. All changes made to the manuscript are indicated in the file titled *Revised Manuscript with Track Changes*.

**Reviewer #1**

**Comment:**

Although the manuscript is an original research, it has a few deficiency. Two things mentioned in the results were not included in the figure;

1) sensitivity analysis after excluding anesthesiologists who performed the most epidural anesthesia and had the highest malposition rate

2) sensitivity analysis after excluding anesthesiologists with the highest range of experience

The information given in the manuscript should also be included in the results.

**Response:**
Thank you for pointing out this inconsistency. We have now included the results of both sensitivity analyses in new *Supplementary Tables (S1,S3 Tables)* rather than in a figure.

Regarding the second analysis, we found that the anesthesiologist with the longest experience (35.4 years) had performed only one epidural anesthesia case. Therefore, instead of excluding only this one case, we conducted a sensitivity analysis by excluding all six cases performed by anesthesiologists with ≥20 years of experience. These results are now presented in S3 Table, and the corresponding description in the Results section has been updated accordingly.

We have revised Figure 2 to display a histogram with postgraduate years grouped in 2-year intervals, which improves the clarity of the distribution of epidural procedures performed by anesthesiologists. Another sensitivity analysis, excluding procedures performed by anesthesiologists with 2.0-2.5 years’ experience, is shown in S2 Table.

**Reviewer #2**

**Comment:**

One negligible spelling mistake: page 4, line 4 reference in brackets not in the right position.

**Response:**
Thank you for pointing this out. We corrected the placement of the reference brackets on page 4, line 4. In addition, we reviewed all other in-text citations throughout the manuscript to ensure that reference numbering and bracket formatting are consistent and accurate.

**Editorial Requirements**

**1. Formatting and File Naming:**
We have revised the manuscript and file names to conform to PLOS ONE’s formatting and style requirements.

**2. Funding Statement:**
We have added the following sentence to clarify the role of the funders in the cover letter:

*“The funders had no role in study design, data collection and analysis, decision to publish, or preparation of the manuscript.”*

**3. Data Availability:**
We confirm that all raw data required to replicate the results of our study are now provided as Supporting Information (S1 Dataset). This includes:

- Data values underlying means and standard deviations
- Raw data used in statistical analyses

We believe this dataset fulfills the PLOS ONE requirement for a minimal data set.

We also re-examined the statistical outputs and found that there were numerical inaccuracies in four specific locations that are not essential to the main conclusions of the manuscript (Lines 163–164, 174, 188, and 191–193). These corrected values are fully traceable and included in the Supporting Information file titled “S1 Dataset.” We carefully verified that all other numerical values in the manuscript are accurate.

**4. Reference List Review:**
We have reviewed all references and confirm that none of the cited papers have been retracted.

We are grateful to the reviewers and the academic editor for their constructive and insightful comments. We hope that the revised manuscript now meets the standards required for publication in *PLOS ONE*.

Sincerely,
Mitsuhiro Matsuo, MD, PhD
On behalf of all authors
